# The role of innate immune signals in immunity to *Brucella abortus*

**DOI:** 10.3389/fcimb.2012.00130

**Published:** 2012-10-25

**Authors:** Marco Túlio R. Gomes, Priscila C. Campos, Leonardo A. de Almeida, Fernanda S. Oliveira, Miriam Maria S. Costa, Fernanda M. Marim, Guilherme S. M. Pereira, Sergio C. Oliveira

**Affiliations:** Department of Biochemistry and Immunology, Institute of Biological Sciences, Federal University of Minas GeraisBelo Horizonte, MG, Brazil

**Keywords:** *Brucella abortus*, innate immunity, type I interferon, TLR signaling, NLR

## Abstract

Innate immunity serves as the first line of defense against infectious agents such as intracellular bacteria. The innate immune platform includes Toll-like receptors (TLRs), retinoid acid-inducible gene-I-like receptors and other cytosolic nucleic acid sensors, nucleotide-binding and oligomerization domain-like receptors, adaptors, kinases and other signaling molecules that are required to elicit effective responses against different pathogens. Our research group has been using the Gram-negative bacteria *Brucella abortus* as a model of pathogen. We have demonstrated that *B. abortus* triggers MAPK and NF-κB signaling pathways in macrophages in a MyD88 and IRAK-4-dependent manner. Furthermore, we claimed that so far TLR9 is the most important single TLR during *Brucella* infection. The identification of host receptors that recognize pathogen-derived nucleic acids has revealed an essential role for nucleic acid sensing in the triggering of immunity to intracellular pathogens. Besides TLRs, herein we describe recent advances in NOD1, NOD2, and type I IFN receptors in innate immune pathways during *B. abortus* infection.

## Introduction

The immune system traditionally has been divided into innate and adaptive that act in an integrated manner to elicit an effective host resistance. The adaptive immune system is slower to develop a response against pathogens when compared to the innate immunity. It responds to specific antigens and generates immunological memory. On the contrary, the innate immune response is rapid and functions as the first line of defense against pathogens. Besides the physical barriers at the surface of the body and the soluble factors, such as complement proteins, it consists mainly of diverse cellular components including macrophages, dendritic cells (DCs), granulocytes (basophils, eosinophils, and neutrophils), and natural killer cells (Dranoff, 2004).

Pathogens regularly display a molecular signature known as pathogens-associated molecular patterns (PAMPs). The most common PAMPs are lipopolysaccharide (LPS), peptidoglycan (PGN), bacterial lipoproteins, flagellin, and nucleic acids derived from viruses, bacteria, fungi, and protozoa. The host innate immune system can interact with these PAMPs using a broad range of germ-line encoded pattern-recognition receptors (PRRs). PRRs comprise the membrane-bound Toll-like receptors (TLRs), which are located either at the cytoplasmic membrane or at the membranes encompassing endosomal vesicles, and sense molecules in the extracellular compartments and endosomal lumen, respectively (Kumar et al., 2011). However, other PRRs can interact with cytosolic stimuli and play an additional role in host-surveillance. The cytosolic RNA-sensing RNA helicases retinoic acid-inducible gene I (RIG-I) and melanoma differentiation-associated gene 5 (MDA5) (Yoneyama et al., 2005); the cytosolic DNA sensors, absent in melanoma 2 (AIM2) and DNA-dependent activator of IRFs (DAI) (Takaoka et al., 2007; Hornung et al., 2009); as well as the nucleotide binding and oligomerization domain (NOD)-like receptors (NLRs) are included in this panoply of intracellular PRRs that recognizes PAMPs and host-derived danger signals (danger-associated molecular patterns, DAMPs) (Schroder and Tschopp, 2010).

Usually, the innate immune cells drive a response against pathogens by sensing molecular stimuli that activate multiple cascades culminating in the expression of an array of genes. Typically, an intact microbial pathogen displays a set of PAMPs that activate multiple PRRs in host cells. This is considered the first step in the activation of multiple intracellular signaling pathways. Hence, signal transduction initiated after the interaction between a PAMP with a specific PRR generally results in the activation of transcription factors. The translocation of activated transcription factors to the cell nucleus ultimately leads to the expression of inflammatory cytokines, type I interferon (IFN), chemokines and other compounds responsible to generate an efficient response against an infection (Bonizzi and Karin, 2004).

The genus *Brucella* is composed of facultative intracellular Gram-negative bacteria that cause brucellosis, a systemic infectious zoonotic disease characterized, among others symptoms, by undulant fever in humans. It was first identified by Sir David Bruce in the 1860s in Malta, which became known as Malta fever (Araj, 2010). The species of *Brucella* are classified according to their preference for specific animal hosts. For instance, the bacterium *B. abortus* naturally infects and causes abortion in cattle, being responsible for severe economical loss (Pappas, 2010). The contacts with infected cattle as well as the consumption of unpasteurized milk from this source are important routes for the transmission of *B. abortus* to humans (Godfroid et al., 2011).

The strategy of *B. abortus* is to evade the innate immune system and persist in the host long enough to be transmitted. The bacterium contains an unusual lipid A composing the LPS molecule, which is important for evading the host immune system during the early stages of infection (Parent et al., 2007). In addition, when entering the host intracellular space, *B. abortus* displays several strategies to avoid the cellular killing mechanism. One of the main virulent factors involved in such action is the Type IV secretion system (T4SS) which is encoded by the *virB* operon (de Jong and Tsolis, 2012). After *B. abortus* is phagocytized, T4SS is induced during phagosomal acidification, leading to the translocation of effector proteins into host cytosol. This process is essential for bacteria subvert lysosome fusion and to create *Brucella*-containing vacuole (BCV), an organelle permissive for replication that interacts with the endoplasmic reticulum (ER) (Celli et al., 2003).

Another strategy employed by *B. abortus* to survive and replicate within macrophages involves host cell death. The attenuated rough *B. abortus* strain RB51 lacks the O-antigen of LPS while the virulent smooth strain S2308 contains the O-antigen portion. It was shown that although RB51 strain induces caspase-2-dependent apoptosis in macrophages, its parental strain 2308 inhibited this programmed cell death mechanism (Chen and He, 2009). Moreover, the apoptosis promoted by rough strains in macrophages was correlated with activation of the NF-κB pathway and production of inflammatory cytokines such as TNF-α (Chen et al., 2011). Interestingly, both S2308 and RB51 induce caspase-2-dependent apoptosis in DC albeit more cell death is promoted by S2308. Taken together, S2308 infection promotes DC apoptosis reducing DC maturation and S2308 antigens presentation to T cells. In addition, the virulent *Brucella* inhibits macrophage cell death avoiding the exposure to the more hostile extracellular environment propitiating the survival and replication inside this cell. In such scenario, the induction of DC cell death and inhibition of macrophage apoptosis might contribute to *Brucella* pathogenesis (Li and He, 2012).

**Figure 1:**
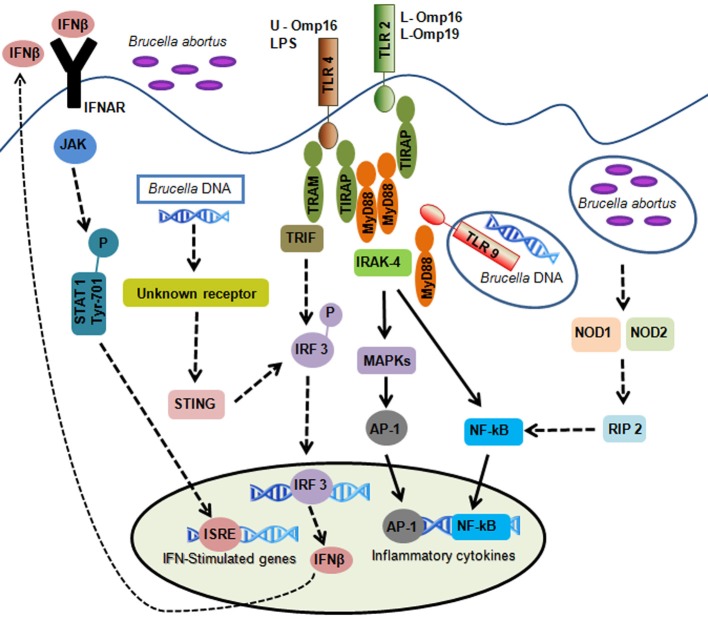
**Overview of innate immune signals during *B. abortus* infection.** *B. abortus*-associated molecular patterns are recognized by pattern-recognition receptors. TLR2 is activated by lipidated outer membrane proteins (L-Omp16 and L-Omp19); TLR4 is activated by *B. abortus* LPS and unlipidated outer membrane protein-16 (U-Omp16); and TLR9 is activated by *B. abortus* DNA. TLR activation leads to intracellular signaling via MyD88 and IRAK-4 resulting in the activation of NF-κB and MAPKs producing inflammatory cytokines. Cytosolic sensors are also involved in *B. abortus* DNA recognition. This pathway involves an unknown receptor leading to IFN-β production dependent on STING and IRF3 activation. Autocrine signaling through the IFN I receptor (IFNAR) leads to activation of signal transducers JAK and STAT1 which culminates in the transcriptional induction of genes that carry promoters with IFN-stimulated response elements (ISRE). Other cytosolic receptors such as NOD1 and NOD2 play an additional role in recognizing *B. abortus*. Activation of these receptors by *Brucella* culminates in NF-κB activation resulting in cytokines synthesis. Solid arrows represent the TLR and non-TLR signaling pathways involving MyD88/IRAK-4 axis important to control *B. abortus* infection. Discontinuous arrows represent TLR, NOD1, NOD2, and type I IFN receptor signaling that are not necessary for *B. abortus* clearance.

Although the stealthy strategy employed by *B. abortus* attempt to create a propitious environment for its replication, host cells are equipped with molecules that interact direct or indirect with this bacterium in order to control the infection. Herein, we will review the prominent findings that have contributed to our understanding of the molecular mechanisms underlying innate immune cell recognition and response to *B. abortus* infection (Figure 1).

## TLR signaling and its role during *Brucella abortus* infection

The TLR family was originally identified in *Drosophila*, where it triggers essential developmental and immunological signaling (Hashimoto et al., 1988). These PRRs were later extensively characterized and their role in innate immunity well established. Currently, 12 members of the TLR family have been identified in mammals being 10 TLRs in humans (TLR1–10). TLR11–13, although less characterized, have been identified in mice, while TLR10 is not expressed in these animals (Kumar et al., 2011). TLRs are type I transmembrane proteins containing three domains: an extracellular domain containing leucine-rich repeats (LRR), which binds PAMPs; a transmembrane domain, spanning the cytoplasmic or endosomal membranes; and an intracellular Toll-Interleukin (IL)-1 receptor (TIR) domain, which interacts with downstream adapter proteins. A common feature of TLRs is to form homo- or heterodimers upon interaction with PAMPs (Kondo et al., 2012).

Based on the cellular localization, TLRs can be divided into two groups. The first group comprises members of TLR family expressed on the cell surface, such as TLR2 which forms heterodimers with TLR1 or TLR6, recognizing bacterial lipopeptides (Kang et al., 2009). Other members that are expressed on cell surface are TLR4 and TLR5, which interacts with bacterial LPS and flagellin, respectively. TLR4 requires the adaptor molecule MD-2 to recognize its ligand. Indeed, MD-2 interacting with TLR4 molecule provides the major LPS binding site, inducing homodimerization of the TLR4–MD-2 complex (Park et al., 2009). Similar to TLR4, upon interaction with its cognate ligand, TLR5 also forms a dimer (Yoon et al., 2012). The second group of TLRs resides in intracellular compartments, with the LRR domains facing organelle lumens and interacting with nucleic acids derived from viral or bacterial pathogens. This group includes TLR3, that recognizes double stranded RNA (dsRNA); TLR7–8, that interacts with single stranded RNA (ssRNA) and TLR9, which binds unmethylated CpG DNA (Chaturvedi and Pierce, 2009; Blasius and Beutler, 2010). Initially, these TLRs are located at ER where they physically interact with UNC93B1. This molecule mediates the translocation of the nucleotide-sensing TLRs from the ER to the endolysosomal compartment, allowing their proper activation by microbial RNA and DNA (Kim et al., 2008).

Activation by PAMPs induces TLRs dimerization which brings the intracellular TIR domains close enough to favor the recruitment of TIR adaptors. This interaction initiates cell signaling leading to transcription factors activation (Zhu and Mohan, 2010; Kawai and Akira, 2011). All TLRs, except TLR3, use the myeloid differentiation factor-88 (MyD88) as adaptor. TLR1, TLR2, TLR4, and TLR6 recruit the TIR domain containing adaptor protein (TIRAP), which acts as a bridge between TLR and MyD88. Activation of TLR3 and TLR4 recruits the TIR domain-containing adapter-inducing IFN-β (TRIF), and TLR4 also requires the adaptor TRIF-related adapter molecule (TRAM) (Kenny and O'Neill, 2008). The recruitment of MyD88 after TLR dimerization is followed by the IL-1 receptor-associated kinase (IRAK) family activation. The IRAKs then recruit the ubiquitin ligase TNF receptor associated (TRAF6) which, in turn, activates TGF-beta-associated kinase 1 (TAK1) (Sorrentino et al., 2008). The TAK1 protein phosphorylates and activates IκB kinase complex (IKK) (Fraczek et al., 2008) and mitogen activated protein kinases (MAPKs), resulting in activation of nuclear factor (NF)-κB (Bhoj and Chen, 2009) and AP-1 (Cargnello and Roux, 2011) transcription factors, respectively. Besides the induction of inflammatory cytokines secretion promoted by TLR7 and TLR9 through NF-κB activation, alternatively these receptors elicit the production of type I IFNs by activating the IFN regulatory factor-7 (IRF-7) (Barber, 2011). Activation of TLR3 involves two distinct pathways initiated by the adaptor TRIF. TRIF recruits TRAF6 resulting in activation of the NF-κB and inflammatory cytokines expression as described above. Alternatively, TRIF binds TANK-binding kinase 1 (TBK-1) activating IRF-3 promotion IFN-β expression (Sato et al., 2003). TLR4 is unique as it recruits four distinct adaptors. The MyD88-dependent pathway involves TIRAP leading to an early-phase activation of NF-κB and AP-1. The MyD88-independent pathway requires the endocytosis of TLR4, which is transported to intracellular vesicles, where it forms a complex with TRAM and TRIF. TRIF then recruits TRAF6 which, in turn, activates TAK1, initiating the late-phase activation of NF-κB and AP-1. TRIF also initiates the signaling cascade to activate IRF-3 through TRAF3 and TBK-1, leading to type I IFN expression (Kawai and Akira, 2011).

Based on the cell signaling initiated upon TLRs activation, it is evident that the protein MyD88 plays a pivotal role in the innate immune response against several pathogens. In fact, it was shown that MyD88^−/−^ mice are inefficient to control *B. abortus* infection and the production of inflammatory cytokines by macrophages is completely abrogated (Weiss et al., 2005). The susceptibility of MyD88^−/−^ mice to *B. abortus* infection was further correlated to impaired DC maturation and lack of IL-12 synthesis by our group (Macedo et al., 2008). Moreover, DC activation during infection stimulates T cells to produce IFN-γ, which plays an essential role controlling *B. abortus* infection (Brandão et al., 2012). IRAK-4, another central protein mediating cell signaling upon TLR activation, was correlated with efficient clearance of *B. abortus* in mice, but not at later phases of infection. Similar to MyD88^−/−^, macrophages and DC derived from IRAK4^−/−^ mice are unable to produce inflammatory cytokines in response to *B. abortus* (Oliveira et al., 2011). The central role attributed to MyD88 and IRAK-4 proteins might suggest the participation of TLRs during the control of *B. abortus* infection. Initially, it was shown that TLR2 plays no role in controlling *B. abortus* infection in mice. Regarding TLR4, there are some controversies in the field. The production of TNF-α by macrophages derived from TLR2^−/−^ and TLR4^−/−^ mice is reduced in *B. abortus* infection. Furthermore, it was shown that the *B. abortus* noncanonical LPS does not display a potent immunostimulatory activity (Weiss et al., 2005). Additionally, it was described a TLR4-dependent production of inflammatory cytokines induced by the unlipidated outer membrane protein (OMP) 16 (U-OMP16) derived from *B. abortus* (Pasquevich et al., 2010). In addition, murine peritoneal macrophages produce TNF-α and IL-6 through TLR2 activation upon infection with *B. abortus* or when stimulated with its lipoprotein L-Omp-19 (Delpino et al., 2012). This result confirms the idea that lipoproteins instead LPS are the main mediators of the inflammatory response elicited by *B. abortus* (Giambartolomei et al., 2004). Thus, TLR2 recognizes *B. abortus* components inducing inflammatory cytokines production. However, this receptor plays no role in mice resistance to *B. abortus* infection *in vivo*. Moreover, TLR9 was correlated with proper *B. abortus* infection control in mice, at least in early stages of infection (at two weeks). Additionally, the production of inflammatory cytokines by DC and macrophages derived from TLR9^−/−^ mice are partially reduced upon stimulation with heat-killed *B. abortus* (HKBa) (Macedo et al., 2008).

The production of immune mediators upon TLR activation depends on intracellular signaling of MAPKs, which include the extracellular signal-regulated kinases 1 and 2 (ERK1/2), c-Jun amino-terminal kinases (JNK) and p38 (Cargnello and Roux, 2011). Previously, it was shown that *B. abortus* rough mutant RB51 and smooth wild type S2308 strain induce phosphorylation of ERK1/2 and p38. However, the activation of these MAPKs is more prominent when the rough strain was used as stimulus (Jiménez De Bagüés et al., 2005). Moreover, our group showed that ERK1/2 and p38 as well as p65 NF-κB phosphorylation is profoundly impaired in IRAK-4^−/−^ and MyD88^−/−^ macrophages activated by *B. abortus* (Oliveira et al., 2011). Clearly, the requirement of MyD88 and IRAK-4 in MAPKs activation argues to the participation of TLRs in cell signaling upon *B. abortus* infection. Indeed, it was shown that HKBa triggers ERK1/2 and p38 phosphorylation through activation of TLR2 in DCs. In that case, p38 but not ERK1/2 activation is correlated with HKBa phagocytosis and IL-12 production (Zhang et al., 2012). Taken together, these findings suggest that *B. abortus* might activate several innate receptors culminating in cell signaling activation and cytokine production.

## NOD like-receptors (NLRs): NOD1, NOD2, and inflammasomes

The NLRs are intracellular molecules that play a critical role in the innate immune system providing surveillance by recognizing structures carried by microbial components at the cytosol (Shaw et al., 2008). NLRs are expressed in immune and non-immune cells such as epithelial and mesothelial cells. There are 23 NLR family members in humans and at least 34 genes in mice (Franchi et al., 2009b). NLRs contain a nucleotide-binding domain (NBD) and a LRR domain. The NBD binds nucleotides inducing conformational changes that are essential to NLRs functions. The LRR domain is required to sense different microbial and endogenous damage stimuli (PAMPs and DAMPs) (Elinav et al., 2011; Franchi et al., 2012). The NLRs display other additional domains that include an amino (N)-terminal caspase recruitment domain (CARD), pyrin domain and acidic transactivating domain or baculovirus inhibitor repeat that mediates downstream protein–protein interaction (Franchi et al., 2012).

The first identified NLRs, NOD1, and NOD2, recognize cell wall fragments from Gram-negative and Gram-positive bacteria. NOD1 senses a specific PGN fragment containing diaminopimelic acid (DAP) that is produced by most Gram-negative and certain Gram-positive bacteria (Chamaillard et al., 2003); while NOD2 is activated by muramyl dipeptide (MDP), which is a conserved structure in practically all types of PGN (Pauleau and Murray, 2003). Following stimulation by their specific bacterial molecules such as DAP or MDP, NOD1, and NOD2 associate with an adaptor molecule, RIP2, through CARD–CARD interaction, which leads to activation of NF-κB and MAPKs promoting induction of numerous genes involved in inflammatory process (Kobayashi et al., 2002; Abbott et al., 2004).

Recent studies described the importance of NOD1 and/or NOD2 signaling in host defense against microbial pathogens such as *Salmonella enterica* (Keestra et al., 2011), *Helicobacter pylori* (Viala et al., 2004), *Listeria monocytogenes* (Kobayashi et al., 2005), *Staphylococcus aureus* (Hruz et al., 2009), and *Legionella pneumophila* (Frutuoso et al., 2010). Although macrophages derived from NOD2-deficient mice were impaired in the production of inflammatory cytokines upon *Mycobacterium tuberculosis* infection, this receptor plays no role controlling the replication of *M. tuberculosis* infection *in vivo* (Gandotra et al., 2007).

Inflammasomes are multiprotein complexes that activate caspase-1 which leads to maturation of the inflammatory cytokines IL-1β and IL-18 and the induction of pyroptosis (Franchi et al., 2009a; Miao et al., 2011). The activation and release of IL-1β and IL-18 requires two signals: the first signal involves the production of inactive precursors (pro-IL-1β and pro-IL-18) and the second signal results in the assembly of the inflammasome and activation of caspase-1 (which might require the adaptor ASC). The activation of caspase-1 is also involved in the induction of pyroptosis, autophagy and pathogen degradation through unidentified mechanisms (Fernandes-Alnemri et al., 2007; Schroder and Tschopp, 2010).

Four major inflammasomes have been identified, three of which belong to NLR family, named after the receptor that regulates their activity: NLR family, pyrin domain-containing 1B (NLRP1B); NLR family, pyrin domain-containing 3 (NLRP3); NLR family, CARD-containing 4 (NLRC4); and AIM2, a receptor of the HIN family of protein (Franchi et al., 2012). Each inflammasome is assembled in a stimulus-specific manner to cluster molecules of pro-caspase-1. For instance, the NLRP1 inflammasome is activated by anthrax lethal toxin and there is some evidence that NLRP1 may detect PGN (Boyden and Dietrich, 2006; Faustin et al., 2007). As for NLRP3, this receptor is activated by a broad range of stimuli, including pathogen-derived signals (bacterial and viral RNA, pore-forming toxins), environment-derived factors (silica, asbestos, alum), and DAMPs (ROS, ATP, uric acid, hyaluronan, amyloid-β) (Kanneganti et al., 2006; Mariathasan et al., 2006; Petrilli et al., 2007; Halle et al., 2008; Hornung et al., 2008; Allen et al., 2009; Martinon et al., 2009; Yamasaki et al., 2009; Eigenbrod et al., 2012). Regarding NLRC4, it senses flagellin or the basal body rod component of the type 3 secretion system found in some bacteria (Miao et al., 2010). The AIM2 inflammasome is a receptor for cytosolic DNA which promotes caspase-1 activation (Hornung et al., 2009; Rathinam et al., 2010). Pyroptosis and production of IL-1β and IL-18 mediated by inflammasomes have been shown to be protective against many infectious agents, including Gram-positive (e.g., *Staphylococcus* and *Listeria*) and Gram-negative (e.g., *Salmonella*, *Legionella*, and *Pseudomonas*) bacteria (Mariathasan et al., 2004, 2006; Lamkanfi and Dixit, 2009; Arlehamn and Evans, 2011; Massis and Zamboni, 2011).

Our group was the first to report the role of the NLRs such as NOD1 and NOD2 during *B. abortus* infection in mice. It was observed a reduced production of TNF-α in bone-marrow derived macrophages from NOD1^−/−^, NOD2^−/−^ and RIP2^−/−^ mice compared to wild-type animals. However, NOD1-, NOD2-, and RIP2-deficient animals showed the same bacterial load in spleens compared to wild-type mice. These results suggest that these molecules may contribute to signal in response to *B. abortus* but they are not essential for host defense against this infection *in vivo* (Oliveira et al., 2012).

## Type I interferon signaling and its role during *Brucella abortus* infection

Classically, exposure of cells to type I IFN induces an antiviral state that prevents productive viral infection. This premise was postulated by Isaacs and Lindenmann about 60 years ago when they demonstrated the cell ability to resist a virus infection. This phenomenon was then attributed to type I IFN cytokine considered the factor responsible to interfere in the viral infection (Isaacs and Lindenmann, 1957). Type I IFN is represented by several partially homologous genes of *IFN-α* and a single gene of *IFN-β* and those genes can be expressed by almost any type of cell in response to stimulation of an array of receptors by pathogens (Decker et al., 2005). The most common and prevalent way by which type I IFN is induced is based on innate immune recognition of PAMPs in cell cytosol. The RIG-I and MDA5 are cytoplasmic receptors responsible for triggering type I IFN secretion after foreign dsRNA recognition transmitting intracellular signaling mediated by the mitochondria-localized adaptor molecule IPS-1 (also known as MAVS) and the kinases TBK1 and IKKi to activate IRF3, the main transcription activator of *IFN-β* gene. Cytosolic DNA sensors such as DAI or Z-DNA binding protein 1 are included in other group of innate immune receptors able to induce type I IFN (Takaoka et al., 2007). However, it was demonstrated that DAI is not the only DNA sensor that activates type I IFN production suggesting a redundant pathway of DNA recognition able to induce IFN. Yang and collaborators (2010) showed that a protein called LRRFIP1 was also able to recognize *Listeria monocytogenes* dsDNA using β-catenin as transcriptional co-activator of *IFN-β* gene. Chiu and colleagues (2009) suggested another intracellular way to recognize foreign DNA. They proposed a mechanism of dsDNA sensing which depends on the ability of cytosolic B-DNA be transcribed into 5′-ppp RNA by RNA-polymerase III, which then activates type I IFN transcription through RIG-I (Chiu et al., 2009). Besides nucleic acid-sensing immune machinery described, TLRs are also considered innate immune receptors able to induce type I IFN in some cells, as described before. For instance, group B streptococcus has been shown to induce type I IFN by conventional DCs via TLR7, MyD88, and IRF1 (Mancuso et al., 2009).

The IFN-β stimulates a classical Janus kinases (Jak1) intracellular pathway through a two subunits receptor called IFN I receptor (IFNAR) (Brierley and Fish, 2002). The subsequent signaling leads to activation of signal transducers (STAT1, STAT2, and IRF9) which culminates in the transcriptional induction of genes that carry promoters with IFN-stimulated response elements (ISRE) such as *IRF7*. The newly synthesized IRF7 translocated to the nucleus when activated binding to *IFNα/β* promoters. In an autocrine and/or paracrine way, IFN-α or IFN-β can act through binding to IFNAR and restart the signaling amplifying the effects of type I IFN (Honda and Taniguchi, 2006). Besides, IFNAR signaling also activates transcriptionally other genes with different action in the cell such as pro-apoptotic genes, chemokines and PKR (dsRNA dependent protein kinase). Regarding that, type I IFN are more than just anti-viral as they play a major role in linking innate to adaptive immunity (Nagarajan et al., 2011).

Besides virus, an increase number of pathogens have been reported to be inducers of type I IFN as bacteria, protozoa, or fungi (Meissner et al., 2005; Monroe et al., 2010; Haque et al., 2011; Sharma et al., 2011; Vivarini Ade et al., 2011). During bacterial infections, type I IFN is often produced, but its effects are conflicting and do not always favoring the immune response of the host against the infection. Type I IFN related to prevent bacterial infection was first demonstrated for pathogens from *Chlamydia* genus (de La Maza et al., 1985). Type I IFN is able to kill the bacteria and inhibit chronic infection due the cytokine effects in blocking the production of infectious organisms since this effect is reversed by exogenous tryptophan and iron which are essential elements for *Chlamydia* growth (Ishihara et al., 2005). The limitation of bacterial infection by type I IFN induction was also observed in the case of *C. pneumoniae* and *Legionella pneumophila* (Plumlee et al., 2009; Buss et al., 2010). The control of *C. pneumoniae* infection in human endothelial cells was dependent of endogenous IFN-β via intracellular signaling dependent of MAVS, IRF3, and IRF7 (Buss et al., 2010). In the case of *L. pneumophila*, the autocrine type I IFN signaling mediated by IFNAR, in a pathway independent of STAT1, -2, and -3, is responsible for bacterial control in the host (Plumlee et al., 2009). Also, type I IFN protects mice against *Salmonella typhimurium* infection due the activation of IFN-γ via STAT4 independent of IL-12 (Freudenberg et al., 2002). Other important group of bacteria that activates IFN I after cellular recognition is group A streptococcus in a intracellular pathway that requires MyD88 but is independent of the TLR2, TLR4, and TLR9 (Gratz et al., 2008). On the other hand, there are a variety of bacteria that are recognized by the innate immune system inducing type I IFN but developing a detrimental phenotype to the host. *Francisella tularensis* and *Listeria monocytogenes* induce type I IFN during infection and it was demonstrated that the signaling mediated by IFNAR is harmful to host since knockout mice for this receptor are more resistant to those infections (Fehr et al., 1997; Auerbuch et al., 2004; Carrero et al., 2004; Stockinger and Decker, 2008; O'Connor et al., 2009; Henry et al., 2010). There are several mechanisms behind type I IFN induction leading to adverse phenotype during bacterial infection. The augmentation of pro-apoptotic stimulus in macrophages and lymphocytes was described in infection with *Listeria, Bacillus anthracis*, *Mycobacteria*, and *Chlamydia* spp and suggested to be related to detrimental phenotype to the host (Carrero et al., 2004; Gold et al., 2004; O'Connell et al., 2004; Decker et al., 2005; Qiu et al., 2008). Even though the pro-apoptotic effects of type I IFN are not fully known, in *L. monocytogenes* infection, type I IFN facilitates the Listeriolysin O-induced lysis of lymphocytes and macrophages facilitating bacteria spread (Carrero et al., 2004, 2006). Type I IFN also is involved in up-regulation of pro-apoptotic innate sensors, such as TLRs and the inflammasomes (Trinchieri, 2010). For instance, type I IFN induced by *Francisella* spp infection augments the expression of AIM2 resulting in caspase-1 activation and in the production of IL-1β and IL-18 as well as inflammasome-mediated cell death in response to cytosolic dsDNA (Choubey et al., 2010; Fernandes-Alnemri et al., 2010). Also, IFN I induced during *F. tularensis* and *L. monocytogenes* infections limits secretion of IL-17 by T cells and limits effective Th1 immune responses to *M. tuberculosis* in part by inducing negative regulator molecules such as suppressor of cytokine signaling proteins (Manca et al., 2005; Henry et al., 2010).

The first observation that *Brucella* induces type I IFN was made by Huang and colleagues (2005) when IFN-α was detected in serum of wild type mice injected with HKBa and the level was markedly reduced in the TLR9^−/−^ mice serum, demonstrating that HKBa induces IFN-α in a TLR9-dependent manner. Additionally, it has shown that *B. abortus* is able to induce IFN-β in DCs (Salcedo et al., 2008). Our group has revealed recently an interesting role of type I IFN during *B. abortus* infection (de Almeida et al., 2011). The absence of type I IFN signaling enhanced host protection to *B. abortus* infection since IFNAR^−/−^ showed to be more resistant to infection than wild type mice. Additionally the spleen derived from *Brucella*-infected IFNAR^−/−^ mice exhibited a drastic reduction in splenic apoptosis compared to wild type controls similar to *L. monocytogenes* results presented by Carrero et al. (2006). Mice lacking IFNAR were more resistant to *B. abortus* infection and displayed less apoptotic lesions as well as reduced expression of pro-apoptotic gene *TRAIL* than their wild type counterparts speculating that type I IFN signaling enhances immune cells apoptosis; therefore, causing the increased susceptibility to *B. abortus*. Furthermore, we demonstrated that *B. abortus* DNA is a major bacterial component to induce IFN-β in macrophages occurring mostly independent of TLRs. Surprisingly, type I IFN expression by *B. abortus* or its DNA was dependent on MyD88, a intracellular pathway observed in *Streptococcus* and *Bacillus anthracis* infection mediated by an unknown PRR (Glomski et al., 2007; Gratz et al., 2008; de Almeida et al., 2011). On the other hand, we demonstrated that the adaptor molecule TRIF played no role in type I IFN induction or *in vivo* host control of *B. abortus*. In addition, we and other groups are intensively searching for cytosolic receptors which are capable of sensing pathogen nucleic acids released from lysed bacteria or from bacterial secretion system during infection (Monroe et al., 2010). Regarding that, we analyzed the potential role of RNA polymerase III in detecting segments of *B. abortus* DNA transcribing them into RNA that induce IFN-β possibly through the RIG-I pathway. Using a inhibitor of RNA polymerase III (ML-60218) (Chiu et al., 2009), we suggested that this enzyme plays a key role in sensing bacterial DNA during *B. abortus* infection triggering intracellular activation of type I IFN. Additionally, the type I IFN induction promoted by *B. abortus* DNA was mediated by an ER resident transmembrane protein termed stimulator of IFN genes (STING) (de Almeida et al., 2011). STING was also identified as essential for type I IFN production in response to *L. monocytogenes* and *C. muridarum* (Ishikawa and Barber, 2008; Prantner et al., 2010). In addition, type I IFN produced in response to *B. abortus* or its DNA signals in an autocrine manner leading to the Tyr701 phosphorylation-dependent activation of STAT1 through IFNAR (de Almeida et al., 2011). In summary, our group has been dissecting host innate response pathways using *B. abortus* as a model of infection. The induction of type I IFN by this bacterium opens up a new avenue to study intracellular pathways induced by pathogens as a way to evade from host immune system.

## Future perspectives

The past decade has witnessed great advances in our understanding of molecular mechanisms underlying innate immune system activation. Structural analysis of several TLRs have elucidated the mechanisms of PAMP recognition by TLR homo- or heterodimers, and many signaling molecules involved in the activation of NF-kB, AP-1, and IRF proteins have been identified and characterized in detail. Regarding *B. abortus* infection, the TLR9 has been suggested so far as the most important single TLR to activate the host immune system. The identification of host receptors that recognize pathogen-derived nucleic acids has revealed an essential role for nucleic acid sensing in the triggering of immunity to intracellular pathogens. Our research group is currently identifying *Brucella* DNA motifs involved in activation of the host innate immune system. Nucleic acids act as adjuvants to activate innate immune programs via TLRs and/or other PRRs and have key roles in facilitating adaptive immunity to intracellular pathogens. Therefore, the use of PAMPs and/or endogenous agonists that stimulate immune cells will be critical for design new effective vaccines. Moreover, during a mammalian infection, *B. abortus* must establish a replicative niche in the presence of a robust innate immune system. The inflammasome is of critical importance to mount an effective innate immune response against intracellular pathogens. Investigation into the interaction of *B. abortus* and the inflammasomes might place a new perspective of sensing bacterial components. A better understanding of the mechanisms underlying *Brucella* interaction with inflammasomes may reveal important findings regarding IL-1β production and its association with the clinical symptoms of intermittent undulant fever, a hallmark of human patients with brucellosis. Understanding the complexity of the transcriptional networks that operate during innate immune receptors activation and define the subsequent immune responses and pathological manifestations during *B. abortus* infection is the major focus of our research.

### Conflict of interest statement

The authors declare that the research was conducted in the absence of any commercial or financial relationships that could be construed as a potential conflict of interest.

## References

[B1] Abbott D. W. Wilkins A. Asara J. M. Cantley L. C. (2004). The Crohn's disease protein, NOD2, requires RIP2 in order to induce ubiquitinylation of a novel site on NEMO. Curr. Biol. 14, 2217–2227 10.1016/j.cub.2004.12.032 15620648

[B2] Allen I. C. Scull M. A. Moore C. B. Holl E. K. McElvania-Tekippe E. Taxman D. J. (2009). The NLRP3 inflammasome mediates *in vivo* innate immunity to influenza A virus through recognition of viral RNA. Immunity 30, 556–565 10.1016/j.immuni.2009.02.005 19362020 PMC2803103

[B3] Araj G. F. (2010). Update on laboratory diagnosis of human brucellosis. Int. J. Antimicrob. Agents 36 Suppl. 1, S12–S17. 10.1016/j.ijantimicag.2010.06.014 20692128

[B4] Arlehamn C. S. Evans T. J. (2011). *Pseudomonas aeruginosa* pilin activates the inflammasome. Cell. Microbiol. 13, 388–401 10.1111/j.1462-5822.2010.01541.x 20955240 PMC3429865

[B5] Auerbuch V. Brockstedt D. G. Meyer-Morse N. O'Riordan M. Portnoy D. A. (2004). Mice lacking the type I interferon receptor are resistant to *Listeria monocytogenes*. J. Exp. Med. 200, 527–533 10.1084/jem.20040976 15302899 PMC2211930

[B6] Barber G. N. (2011). Innate immune DNA sensing pathways: STING, AIMII and the regulation of interferon production and inflammatory responses. Curr. Opin. Immunol. 23, 10–20 10.1016/j.coi.2010.12.015 21239155 PMC3881186

[B7] Bhoj V. G. Chen Z. J. (2009). Ubiquitylation in innate and adaptive immunity. Nature 458, 430–437 10.1038/nature07959 19325622

[B8] Blasius A. L. Beutler B. (2010). Intracellular toll-like receptors. Immunity 32, 305–315 10.1016/j.immuni.2010.03.012 20346772

[B9] Bonizzi G. Karin M. (2004). The two NF-kappaB activation pathways and their role in innate and adaptive immunity. Trends Immunol. 25, 280–288 10.1016/j.it.2004.03.008 15145317

[B10] Boyden E. D. Dietrich W. F. (2006). Nalp1b controls mouse macrophage susceptibility to anthrax lethal toxin. Nat. Genet. 38, 240–244 10.1038/ng1724 16429160

[B11] Brandão A. P. Oliveira F. S. Carvalho N. B. Vieira L. Q. Azevedo V. Macedo G. C. (2012). Host susceptibility to *Brucella abortus* infection is more pronounced in IFN-gamma knockout than IL-12/beta2-microglobulin double-deficient mice. Clin. Dev. Immunol. 2012, 589494 22194770 10.1155/2012/589494 PMC3238360

[B12] Brierley M. M. Fish E. N. (2002). Review: IFN-alpha/beta receptor interactions to biologic outcomes: understanding the circuitry. J. Interferon Cytokine Res. 22, 835–845 10.1089/107999002760274845 12396722

[B13] Buss C. Opitz B. Hocke A. C. Lippmann J. Van Laak V. Hippenstiel S. (2010). Essential role of mitochondrial antiviral signaling, IFN regulatory factor (IRF)3, and IRF7 in *Chlamydophila pneumoniae*-mediated IFN-beta response and control of bacterial replication in human endothelial cells. J. Immunol. 184, 3072–3078 10.4049/jimmunol.0902947 20154210

[B14] Cargnello M. Roux P. P. (2011). Activation and function of the MAPKs and their substrates, the MAPK-activated protein kinases. Microbiol. Mol. Biol. Rev. 75, 50–83 10.1128/MMBR.00031-10 21372320 PMC3063353

[B15] Carrero J. A. Calderon B. Unanue E. R. (2004). Type I interferon sensitizes lymphocytes to apoptosis and reduces resistance to Listeria infection. J. Exp. Med. 200, 535–540 10.1084/jem.20040769 15302900 PMC2211931

[B16] Carrero J. A. Calderon B. Unanue E. R. (2006). Lymphocytes are detrimental during the early innate immune response against *Listeria monocytogenes*. J. Exp. Med. 203, 933–940 10.1084/jem.20060045 16549598 PMC2118284

[B17] Celli J. De Chastellier C. Franchini D. M. Pizarro-Cerda J. Moreno E. Gorvel J. P. (2003). *Brucella* evades macrophage killing via VirB-dependent sustained interactions with the endoplasmic reticulum. J. Exp. Med. 198, 545–556 10.1084/jem.20030088 12925673 PMC2194179

[B18] Chamaillard M. Hashimoto M. Horie Y. Masumoto J. Qiu S. Saab L. (2003). An essential role for NOD1 in host recognition of bacterial peptidoglycan containing diaminopimelic acid. Nat. Immunol. 4, 702–707 10.1038/ni945 12796777

[B19] Chaturvedi A. Pierce S. K. (2009). How location governs toll-like receptor signaling. Traffic 10, 621–628 10.1111/j.1600-0854.2009.00899.x 19302269 PMC2741634

[B20] Chen F. Ding X. Ding Y. Xiang Z. Li X. Ghosh D. (2011). Proinflammatory caspase-2-mediated macrophage cell death induced by a rough attenuated *Brucella suis* strain. Infect. Immun. 79, 2460–2469 10.1128/IAI.00050-11 21464087 PMC3125819

[B21] Chen F. He Y. (2009). Caspase-2 mediated apoptotic and necrotic murine macrophage cell death induced by rough Brucella abortus. PLoS ONE 4:e6830 10.1371/journal.pone.0006830 19714247 PMC2729395

[B22] Chiu Y. H. Macmillan J. B. Chen Z. J. (2009). RNA polymerase III detects cytosolic DNA and induces type I interferons through the RIG-I pathway. Cell 138, 576–591 10.1016/j.cell.2009.06.015 19631370 PMC2747301

[B23] Choubey D. Duan X. Dickerson E. Ponomareva L. Panchanathan R. Shen H. (2010). Interferon-inducible p200-family proteins as novel sensors of cytoplasmic DNA: role in inflammation and autoimmunity. J. Interferon Cytokine Res. 30, 371–380 10.1089/jir.2009.0096 20187776 PMC2947464

[B24] de Almeida L. A. Carvalho N. B. Oliveira F. S. Lacerda T. L. Vasconcelos A. C. Nogueira L. (2011). MyD88 and STING signaling pathways are required for IRF3-mediated IFN-beta induction in response to *Brucella abortus* infection. PLoS ONE 6:e23135 10.1371/journal.pone.0023135 21829705 PMC3149075

[B25] de Jong M. F. Tsolis R. M. (2012). Brucellosis and type IV secretion. Future Microbiol. 7, 47–58 10.2217/fmb.11.136 22191446

[B26] de La Maza L. M. Peterson E. M. Goebel J. M. Fennie C. W. Czarniecki C. W. (1985). Interferon-induced inhibition of *Chlamydia trachomatis*: dissociation from antiviral and antiproliferative effects. Infect. Immun. 47, 719–722 3972450 10.1128/iai.47.3.719-722.1985 PMC261369

[B27] Decker T. Muller M. Stockinger S. (2005). The yin and yang of type I interferon activity in bacterial infection. Nat. Rev. Immunol. 5, 675–687 10.1038/nri1684 16110316

[B28] Delpino M. V. Barrionuevo P. Macedo G. C. Oliveira S. C. Genaro S. D. Scian R. (2012). Macrophage-elicited osteoclastogenesis in response to *Brucella abortus* infection requires TLR2/MyD88-dependent TNF-alpha production. J. Leukoc. Biol. 91, 285–298 10.1189/jlb.04111185 22075930

[B29] Dranoff G. (2004). Cytokines in cancer pathogenesis and cancer therapy. Nat. Rev. Cancer 4, 11–22 10.1038/nrc1252 14708024

[B30] Eigenbrod T. Franchi L. Munoz-Planillo R. Kirschning C. J. Freudenberg M. A. Nunez G. (2012). Bacterial RNA mediates activation of caspase-1 and IL-1beta release independently of TLRs 3 7, 9 and TRIF but Is dependent on UNC93B. J. Immunol. 189, 328–336 10.4049/jimmunol.1103258 22634614 PMC4327882

[B31] Elinav E. Strowig T. Henao-Mejia J. Flavell R. A. (2011). Regulation of the antimicrobial response by NLR proteins. Immunity 34, 665–679 10.1016/j.immuni.2011.05.007 21616436

[B32] Faustin B. Lartigue L. Bruey J. M. Luciano F. Sergienko E. Bailly-Maitre B. (2007). Reconstituted NALP1 inflammasome reveals two-step mechanism of caspase-1 activation. Mol. Cell 25, 713–724 10.1016/j.molcel.2007.01.032 17349957

[B33] Fehr T. Schoedon G. Odermatt B. Holtschke T. Schneemann M. Bachmann M. F. (1997). Crucial role of interferon consensus sequence binding protein, but neither of interferon regulatory factor 1 nor of nitric oxide synthesis for protection against murine listeriosis. J. Exp. Med. 185, 921–931 10.1084/jem.185.5.921 9120398 PMC2196174

[B34] Fernandes-Alnemri T. Wu J. Yu J. W. Datta P. Miller B. Jankowski W. (2007). The pyroptosome: a supramolecular assembly of ASC dimers mediating inflammatory cell death via caspase-1 activation. Cell Death Differ. 14, 1590–1604 10.1038/sj.cdd.4402194 17599095 PMC3345951

[B35] Fernandes-Alnemri T. Yu J. W. Juliana C. Solorzano L. Kang S. Wu J. (2010). The AIM2 inflammasome is critical for innate immunity to *Francisella tularensis*. Nat. Immunol. 11, 385–393 10.1038/ni.1859 20351693 PMC3111085

[B36] Fraczek J. Kim T. W. Xiao H. Yao J. Wen Q. Li Y. (2008). The kinase activity of IL-1 receptor-associated kinase 4 is required for interleukin-1 receptor/toll-like receptor-induced TAK1-dependent NFkappaB activation. J. Biol. Chem. 283, 31697–31705 10.1074/jbc.M804779200 18794297 PMC2581573

[B37] Franchi L. Eigenbrod T. Munoz-Planillo R. Nunez G. (2009a). The inflammasome: a caspase-1-activation platform that regulates immune responses and disease pathogenesis. Nat. Immunol. 10, 241–247 10.1038/ni.1703 19221555 PMC2820724

[B39] Franchi L. Warner N. Viani K. Nunez G. (2009b). Function of Nod-like receptors in microbial recognition and host defense. Immunol. Rev. 227, 106–128 10.1111/j.1600-065X.2008.00734.x 19120480 PMC2679989

[B38] Franchi L. Munoz-Planillo R. Nunez G. (2012). Sensing and reacting to microbes through the inflammasomes. Nat. Immunol. 13, 325–332 10.1038/ni.2231 22430785 PMC3449002

[B40] Freudenberg M. A. Merlin T. Kalis C. Chvatchko Y. Stubig H. Galanos C. (2002). Cutting edge: a murine, IL-12-independent pathway of IFN-gamma induction by gram-negative bacteria based on STAT4 activation by Type I IFN and IL-18 signaling. J. Immunol. 169, 1665–1668 12165484 10.4049/jimmunol.169.4.1665

[B41] Frutuoso M. S. Hori J. I. Pereira M. S. Junior D. S. Sonego F. Kobayashi K. S. (2010). The pattern recognition receptors Nod1 and Nod2 account for neutrophil recruitment to the lungs of mice infected with *Legionella pneumophila*. Microbes Infect. 12, 819–827 10.1016/j.micinf.2010.05.006 20685341

[B42] Gandotra S. Jang S. Murray P. J. Salgame P. Ehrt S. (2007). Nucleotide-binding oligomerization domain protein 2-deficient mice control infection with *Mycobacterium tuberculosis*. Infect. Immun. 75, 5127–5134 10.1128/IAI.00458-07 17709422 PMC2168277

[B43] Giambartolomei G. H. Zwerdling A. Cassataro J. Bruno L. Fossati C. A. Philipp M. T. (2004). Lipoproteins, not lipopolysaccharide, are the key mediators of the proinflammatory response elicited by heat-killed *Brucella abortus*. J. Immunol. 173, 4635–4642 15383598 10.4049/jimmunol.173.7.4635

[B44] Glomski I. J. Fritz J. H. Keppler S. J. Balloy V. Chignard M. Mock M. (2007). Murine splenocytes produce inflammatory cytokines in a MyD88-dependent response to Bacillus anthracis spores. Cell. Microbiol. 9, 502–513 10.1111/j.1462-5822.2006.00806.x 16978234

[B45] Godfroid J. Scholz H. C. Barbier T. Nicolas C. Wattiau P. Fretin D. (2011). Brucellosis at the animal/ecosystem/human interface at the beginning of the 21st century. Prev. Vet. Med. 102, 118–131 10.1016/j.prevetmed.2011.04.007 21571380

[B46] Gold J. A. Hoshino Y. Hoshino S. Jones M. B. Nolan A. Weiden M. D. (2004). Exogenous gamma and alpha/beta interferon rescues human macrophages from cell death induced by *Bacillus anthracis*. Infect. Immun. 72, 1291–1297 10.1128/IAI.72.3.1291-1297.2004 14977930 PMC356021

[B47] Gratz N. Siller M. Schaljo B. Pirzada Z. A. Gattermeier I. Vojtek I. (2008). Group A streptococcus activates type I interferon production and MyD88-dependent signaling without involvement of TLR2, TLR4, and TLR9. J. Biol. Chem. 283, 19879–19887 10.1074/jbc.M802848200 18480050 PMC2459277

[B48] Halle A. Hornung V. Petzold G. C. Stewart C. R. Monks B. G. Reinheckel T. (2008). The NALP3 inflammasome is involved in the innate immune response to amyloid-beta. Nat. Immunol. 9, 857–865 10.1038/ni.1636 18604209 PMC3101478

[B49] Haque A. Best S. E. Ammerdorffer A. Desbarrieres L. De Oca M. M. Amante F. H. (2011). Type I interferons suppress CD4(+) T-cell-dependent parasite control during blood-stage *Plasmodium* infection. Eur. J. Immunol. 41, 2688–2698 10.1002/eji.201141539 21674481

[B50] Hashimoto C. Hudson K. L. Anderson K. V. (1988). The Toll gene of *Drosophila*, required for dorsal-ventral embryonic polarity, appears to encode a transmembrane protein. Cell 52, 269–279 10.1016/0092-8674(88)90516-8 2449285

[B51] Henry T. Kirimanjeswara G. S. Ruby T. Jones J. W. Peng K. Perret M. (2010). Type I IFN signaling constrains IL-17A/F secretion by gammadelta T cells during bacterial infections. J. Immunol. 184, 3755–3767 10.4049/jimmunol.0902065 20176744 PMC2879132

[B52] Honda K. Taniguchi T. (2006). IRFs: master regulators of signalling by Toll-like receptors and cytosolic pattern-recognition receptors. Nat. Rev. Immunol. 6, 644–658 10.1038/nri1900 16932750

[B53] Hornung V. Ablasser A. Charrel-Dennis M. Bauernfeind F. Horvath G. Caffrey D. R. (2009). AIM2 recognizes cytosolic dsDNA and forms a caspase-1-activating inflammasome with ASC. Nature 458, 514–518 10.1038/nature07725 19158675 PMC2726264

[B54] Hornung V. Bauernfeind F. Halle A. Samstad E. O. Kono H. Rock K. L. (2008). Silica crystals and aluminum salts activate the NALP3 inflammasome through phagosomal destabilization. Nat. Immunol. 9, 847–856 10.1038/ni.1631 18604214 PMC2834784

[B55] Hruz P. Zinkernagel A. S. Jenikova G. Botwin G. J. Hugot J. P. Karin M. (2009). NOD2 contributes to cutaneous defense against *Staphylococcus aureus* through alpha-toxin-dependent innate immune activation. Proc. Natl. Acad. Sci. U.S.A. 106, 12873–12878 10.1073/pnas.0904958106 19541630 PMC2722361

[B56] Huang L. Y. Ishii K. J. Akira S. Aliberti J. Golding B. (2005). Th1-like cytokine induction by heat-killed *Brucella abortus* is dependent on triggering of TLR9. J. Immunol. 175, 3964–3970 16148144 10.4049/jimmunol.175.6.3964

[B57] Isaacs A. Lindenmann J. (1957). Virus interference. I. The interferon. Proc. R. Soc. Lond. B Biol. Sci. 147, 258–267 10.1016/j.biochi.2007.01.005 26297790

[B58] Ishihara T. Aga M. Hino K. Ushio C. Taniguchi M. Iwaki K. (2005). Inhibition of *Chlamydia trachomatis* growth by human interferon-alpha: mechanisms and synergistic effect with interferon-gamma and tumor necrosis factor-alpha. Biomed. Res. 26, 179–185 16152734 10.2220/biomedres.26.179

[B59] Ishikawa H. Barber G. N. (2008). STING is an endoplasmic reticulum adaptor that facilitates innate immune signalling. Nature 455, 674–678 10.1038/nature07317 18724357 PMC2804933

[B60] Jiménez De Bagüés M. P. Gross A. Terraza A. Dornand J. (2005). Regulation of the mitogen-activated protein kinases by *Brucella* spp. expressing a smooth and rough phenotype: relationship to pathogen invasiveness. Infect. Immun. 73, 3178–3183 10.1128/IAI.73.5.3178-3183.2005 15845529 PMC1087367

[B61] Kang J. Y. Nan X. Jin M. S. Youn S. J. Ryu Y. H. Mah S. (2009). Recognition of lipopeptide patterns by Toll-like receptor 2-Toll-like receptor 6 heterodimer. Immunity 31, 873–884 10.1016/j.immuni.2009.09.018 19931471

[B62] Kanneganti T. D. Ozoren N. Body-Malapel M. Amer A. Park J. H. Franchi L. (2006). Bacterial RNA and small antiviral compounds activate caspase-1 through cryopyrin/Nalp3. Nature 440, 233–236 10.1038/nature04517 16407888

[B63] Kawai T. Akira S. (2011). Toll-like receptors and their crosstalk with other innate receptors in infection and immunity. Immunity 34, 637–650 10.1016/j.immuni.2011.05.006 21616434

[B64] Keestra A. M. Winter M. G. Klein-Douwel D. Xavier M. N. Winter S. E. Kim A. (2011). A *Salmonella* virulence factor activates the NOD1/NOD2 signaling pathway. MBio 2, 266–276 10.1128/mBio.00266-11 22186610 PMC3238304

[B65] Kenny E. F. O'Neill L. A. (2008). Signalling adaptors used by Toll-like receptors: an update. Cytokine 43, 342–349 10.1016/j.cyto.2008.07.010 18706831

[B66] Kim Y. M. Brinkmann M. M. Paquet M. E. Ploegh H. L. (2008). UNC93B1 delivers nucleotide-sensing toll-like receptors to endolysosomes. Nature 452, 234–238 10.1038/nature06726 18305481

[B67] Kobayashi K. Inohara N. Hernandez L. D. Galan J. E. Nunez G. Janeway C. A. (2002). RICK/Rip2/CARDIAK mediates signalling for receptors of the innate and adaptive immune systems. Nature 416, 194–199 10.1038/416194a 11894098

[B68] Kobayashi K. S. Chamaillard M. Ogura Y. Henegariu O. Inohara N. Nuñez G. (2005). Nod2-dependent regulation of innate and adaptive immunity in the intestinal tract. Science 307, 731–734 10.1126/science.1104911 15692051

[B69] Kondo T. Kawai T. Akira S. (2012). Dissecting negative regulation of Toll-like receptor signaling. Trends Immunol. 33, 449–458 10.1016/j.it.2012.05.002 22721918

[B70] Kumar H. Kawai T. Akira S. (2011). Pathogen recognition by the innate immune system. Int. Rev. Immunol. 30, 16–34 10.3109/08830185.2010.529976 21235323

[B71] Lamkanfi M. Dixit V. M. (2009). The inflammasomes. PLoS Pathog. 5:e1000510 10.1371/journal.ppat.1000510 20041168 PMC2791419

[B72] Li X. He Y. (2012). Caspase-2-dependent dendritic cell death, maturation, and priming of T cells in response to *Brucella abortus* infection. PLoS ONE 7:e43512 10.1371/journal.pone.0043512 22927979 PMC3425542

[B73] Macedo G. C. Magnani D. M. Carvalho N. B. Bruna-Romero O. Gazzinelli R. T. Oliveira S. C. (2008). Central role of MyD88-dependent dendritic cell maturation and proinflammatory cytokine production to control *Brucella abortus* infection. J. Immunol. 180, 1080–1087 18178848 10.4049/jimmunol.180.2.1080

[B74] Manca C. Tsenova L. Freeman S. Barczak A. K. Tovey M. Murray P. J. (2005). Hypervirulent, *M*. *tuberculosis* W/Beijing strains upregulate type I IFNs and increase expression of negative regulators of the Jak-Stat pathway. J. Interferon Cytokine Res. 25, 694–701 10.1089/jir.2005.25.694 16318583

[B75] Mancuso G. Gambuzza M. Midiri A. Biondo C. Papasergi S. Akira S. (2009). Bacterial recognition by TLR7 in the lysosomes of conventional dendritic cells. Nat. Immunol. 10, 587–594 10.1038/ni.1733 19430477

[B76] Mariathasan S. Newton K. Monack D. M. Vucic D. French D. M. Lee W. P. (2004). Differential activation of the inflammasome by caspase-1 adaptors ASC and Ipaf. Nature 430, 213–218 10.1038/nature02664 15190255

[B77] Mariathasan S. Weiss D. S. Newton K. McBride J. O'Rourke K. Roose-Girma M. Lee W. P. (2006). Cryopyrin activates the inflammasome in response to toxins and ATP. Nature 440, 228–232 10.1038/nature04515 16407890

[B78] Martinon F. Mayor A. Tschopp J. (2009). The inflammasomes: guardians of the body. Annu. Rev. Immunol. 27, 229–265 10.1146/annurev.immunol.021908.132715 19302040

[B79] Massis L. M. Zamboni D. S. (2011). Innate immunity to *Legionella pneumophila*. Front. Microbiol. 2:109 10.3389/fmicb.2011.00109 21833338 PMC3153058

[B80] Meissner N. N. Swain S. Tighe M. Harmsen A. Harmsen A. (2005). Role of type I IFNs in pulmonary complications of *Pneumocystis murina* infection. J. Immunol. 174, 5462–5471 15843544 10.4049/jimmunol.174.9.5462

[B81] Miao E. A. Mao D. P. Yudkovsky N. Bonneau R. Lorang C. G. Warren S. E. (2010). Innate immune detection of the type III secretion apparatus through the NLRC4 inflammasome. Proc. Natl. Acad. Sci. U.S.A. 107, 3076–3080 10.1073/pnas.0913087107 20133635 PMC2840275

[B82] Miao E. A. Rajan J. V. Aderem A. (2011). Caspase-1-induced pyroptotic cell death. Immunol. Rev. 243, 206–214 10.1111/j.1600-065X.2011.01044.x 21884178 PMC3609431

[B83] Monroe K. M. McWhirter S. M. Vance R. E. (2010). Induction of type I interferons by bacteria. Cell. Microbiol. 12, 881–890 10.1111/j.1462-5822.2010.01478.x 20482555 PMC2897911

[B84] Nagarajan U. M. Sikes J. Prantner D. Andrews C. W. Jr. Frazer L. Goodwin A. (2011). MyD88 deficiency leads to decreased NK cell gamma interferon production and T cell recruitment during *Chlamydia muridarum* genital tract infection, but a predominant Th1 response and enhanced monocytic inflammation are associated with infection resolution. Infect. Immun. 79, 486–498 10.1128/IAI.00843-10 21078858 PMC3019903

[B85] O'Connell R. M. Saha S. K. Vaidya S. A. Bruhn K. W. Miranda G. A. Zarnegar B. (2004). Type I interferon production enhances susceptibility to *Listeria monocytogenes* infection. J. Exp. Med. 200, 437–445 10.1084/jem.20040712 15302901 PMC2211937

[B86] O'Connor W. Jr. Kamanaka M. Booth C. J. Town T. Nakae S. Iwakura Y. (2009). A protective function for interleukin 17A in T cell-mediated intestinal inflammation. Nat. Immunol. 10, 603–609 10.1038/ni.1736 19448631 PMC2709990

[B87] Oliveira F. S. Carvalho N. B. Brandao A. P. Gomes M. T. De Almeida L. A. Oliveira S. C. (2011). Interleukin-1 receptor-associated kinase 4 is essential for initial host control of *Brucella abortus* infection. Infect. Immun. 79, 4688–4695 10.1128/IAI.05289-11 21844234 PMC3257947

[B88] Oliveira F. S. Carvalho N. B. Zamboni D. S. Oliveira S. C. (2012). Nucleotide-binding oligomerization domain-1 and -2 play no role in controlling *Brucella abortus* infection in mice. Clin. Dev. Immunol. 2012, 861426 10.1155/2012/861426 22203860 PMC3235452

[B89] Pappas G. (2010). The changing *Brucella* ecology: novel reservoirs, new threats. Int. J. Antimicrob. Agents 36 Suppl. 1, S8–S11 10.1016/j.ijantimicag.2010.06.013 20696557

[B90] Parent M. A. Goenka R. Murphy E. Levier K. Carreiro N. Golding B. (2007). *Brucella abortus* bacA mutant induces greater pro-inflammatory cytokines than the wild-type parent strain. Microbes Infect. 9, 55–62 10.1016/j.micinf.2006.10.008 17196866

[B91] Park B. S. Song D. H. Kim H. M. Choi B. S. Lee H. Lee J. O. (2009). The structural basis of lipopolysaccharide recognition by the TLR4-MD-2 complex. Nature 458, 1191–1195 10.1038/nature07830 19252480

[B92] Pasquevich K. A. Garcia Samartino C. Coria L. M. Estein S. M. Zwerdling A. Ibanez A. E. (2010). The protein moiety of *Brucella abortus* outer membrane protein 16 is a new bacterial pathogen-associated molecular pattern that activates dendritic cells *in vivo*, induces a Th1 immune response, and is a promising self-adjuvanting vaccine against systemic and oral acquired brucellosis. J. Immunol. 184, 5200–5212 10.4049/jimmunol.0902209 20351187

[B93] Pauleau A. L. Murray P. J. (2003). Role of nod2 in the response of macrophages to toll-like receptor agonists. Mol. Cell. Biol. 23, 7531–7539 10.1128/MCB.23.21.7531-7539.2003 14560001 PMC207570

[B94] Petrilli V. Papin S. Dostert C. Mayor A. Martinon F. Tschopp J. (2007). Activation of the NALP3 inflammasome is triggered by low intracellular potassium concentration. Cell Death Differ. 14, 1583–1589 10.1038/sj.cdd.4402195 17599094

[B95] Plumlee C. R. Lee C. Beg A. A. Decker T. Shuman H. A. Schindler C. (2009). Interferons direct an effective innate response to *Legionella pneumophila* infection. J. Biol. Chem. 284, 30058–30066 10.1074/jbc.M109.018283 19720834 PMC2781560

[B96] Prantner D. Darville T. Nagarajan U. M. (2010). Stimulator of IFN gene is critical for induction of IFN-beta during *Chlamydia muridarum* infection. J. Immunol. 184, 2551–2560 10.4049/jimmunol.0903704 20107183 PMC2863030

[B97] Qiu H. Fan Y. Joyee A. G. Wang S. Han X. Bai H. (2008). Type I IFNs enhance susceptibility to *Chlamydia muridarum* lung infection by enhancing apoptosis of local macrophages. J. Immunol. 181, 2092–2102 18641348 10.4049/jimmunol.181.3.2092

[B98] Rathinam V. A. Jiang Z. Waggoner S. N. Sharma S. Cole L. E. Waggoner L. (2010). The AIM2 inflammasome is essential for host defense against cytosolic bacteria and DNA viruses. Nat. Immunol. 11, 395–402 10.1038/ni.1864 20351692 PMC2887480

[B99] Salcedo S. P. Marchesini M. I. Lelouard H. Fugier E. Jolly G. Balor S. (2008). *Brucella* control of dendritic cell maturation is dependent on the TIR-containing protein Btp1. PLoS Pathog. 4:e21 10.1371/journal.ppat.0040021 18266466 PMC2233671

[B100] Sato S. Sugiyama M. Yamamoto M. Watanabe Y. Kawai T. Takeda K. (2003). Toll/IL-1 receptor domain-containing adaptor inducing IFN-beta (TRIF) associates with TNF receptor-associated factor 6 and TANK-binding kinase 1, and activates two distinct transcription factors, NF-kappa B and IFN-regulatory factor-3, in the Toll-like receptor signaling. J. Immunol. 171, 4304–4310 14530355 10.4049/jimmunol.171.8.4304

[B101] Schroder K. Tschopp J. (2010). The inflammasomes. Cell 140, 821–832 10.1016/j.cell.2010.01.040 20303873

[B102] Sharma S. Deoliveira R. B. Kalantari P. Parroche P. Goutagny N. Jiang Z. (2011). Innate immune recognition of an AT-rich stem-loop DNA motif in the *Plasmodium falciparum* genome. Immunity 35, 194–207 10.1016/j.immuni.2011.05.016 21820332 PMC3162998

[B103] Shaw M. H. Reimer T. Kim Y. G. Nunez G. (2008). NOD-like receptors (NLRs): bona fide intracellular microbial sensors. Curr. Opin. Immunol. 20, 377–382 10.1016/j.coi.2008.06.001 18585455 PMC2572576

[B104] Sorrentino A. Thakur N. Grimsby S. Marcusson A. Von Bulow V. Schuster N. (2008). The type I TGF-beta receptor engages TRAF6 to activate TAK1 in a receptor kinase-independent manner. Nat. Cell Biol. 10, 1199–1207 10.1038/ncb1780 18758450

[B105] Stockinger S. Decker T. (2008). Novel functions of type I interferons revealed by infection studies with *Listeria monocytogenes*. Immunobiology 213, 889–897 10.1016/j.imbio.2008.07.020 18926303

[B106] Takaoka A. Wang Z. Choi M. K. Yanai H. Negishi H. Ban T. (2007). DAI (DLM-1/ZBP1) is a cytosolic DNA sensor and an activator of innate immune response. Nature 448, 501–505 10.1038/nature06013 17618271

[B107] Trinchieri G. (2010). Type I interferon: friend or foe? J. Exp. Med. 207, 2053–2063 10.1084/jem.20101664 20837696 PMC2947062

[B108] Viala J. Chaput C. Boneca I. G. Cardona A. Girardin S. E. Moran A. P. (2004). Nod1 responds to peptidoglycan delivered by the *Helicobacter pylori* cag pathogenicity island. Nat. Immunol. 5, 1166–1174 10.1038/ni1131 15489856

[B109] Vivarini Ade C. Pereira Rde M. Teixeira K. L. Calegari-Silva T. C. Bellio M. Laurenti M. D. (2011). Human cutaneous leishmaniasis: interferon-dependent expression of double-stranded RNA-dependent protein kinase (PKR) via TLR2. FASEB J. 25, 4162–4173 10.1096/fj.11-185165 21846836

[B110] Weiss D. S. Takeda K. Akira S. Zychlinsky A. Moreno E. (2005). MyD88, but not toll-like receptors 4 and 2, is required for efficient clearance of *Brucella abortus*. Infect. Immun. 73, 5137–5143 10.1128/IAI.73.8.5137-5143.2005 16041030 PMC1201196

[B111] Yamasaki K. Muto J. Taylor K. R. Cogen A. L. Audish D. Bertin J. (2009). NLRP3/cryopyrin is necessary for interleukin-1beta (IL-1beta) release in response to hyaluronan, an endogenous trigger of inflammation in response to injury. J. Biol. Chem. 284, 12762–12771 10.1074/jbc.M806084200 19258328 PMC2676006

[B112] Yang P. An H. Liu X. Wen M. Zheng Y. Rui Y. (2010). The cytosolic nucleic acid sensor LRRFIP1 mediates the production of type I interferon via a beta-catenin-dependent pathway. Nat. Immunol. 11, 487–494 10.1038/ni.1876 20453844

[B113] Yoneyama M. Kikuchi M. Matsumoto K. Imaizumi T. Miyagishi M. Taira K. (2005). Shared and unique functions of the DExD/H-box helicases RIG-I, MDA5, and LGP2 in antiviral innate immunity. J. Immunol. 175, 2851–2858 16116171 10.4049/jimmunol.175.5.2851

[B114] Yoon S. I. Kurnasov O. Natarajan V. Hong M. Gudkov A. V. Osterman A. L. (2012). Structural basis of TLR5-flagellin recognition and signaling. Science 335, 859–864 10.1126/science.1215584 22344444 PMC3406927

[B115] Zhang C. Y. Bai N. Zhang Z. H. Liang N. Dong L. Xiang R. (2012). TLR2 signaling subpathways regulate TLR9 signaling for the effective induction of IL-12 upon stimulation by heat-killed *Brucella abortus*. Cell. Mol. Immunol. 9, 324–333 10.1038/cmi.2012.11 22635254 PMC4012865

[B116] Zhu J. Mohan C. (2010). Toll-like receptor signaling pathways–therapeutic opportunities. Mediators Inflamm. 2010, 781235 10.1155/2010/781235 20981241 PMC2963142

